# Compressed Sensing for fMRI: Feasibility Study on the Acceleration of Non-EPI fMRI at 9.4T

**DOI:** 10.1155/2015/131926

**Published:** 2015-08-27

**Authors:** Paul Kyu Han, Sung-Hong Park, Seong-Gi Kim, Jong Chul Ye

**Affiliations:** ^1^Bio Imaging and Signal Processing Lab, Department of Bio and Brain Engineering, Korea Advanced Institute of Science and Technology (KAIST), 373-1 Guseong-dong, Yuseong-gu, Daejeon 305-701, Republic of Korea; ^2^Magnetic Resonance Imaging Lab, Department of Bio and Brain Engineering, Korea Advanced Institute of Science and Technology (KAIST), 373-1 Guseong-dong, Yuseong-gu, Daejeon 305-701, Republic of Korea; ^3^Center for Neuroscience Imaging Research, Institute for Basic Science (IBS), Suwon 440-746, Republic of Korea; ^4^Department of Biomedical Engineering, Sungkyunkwan University (SKKU), Suwon 440-746, Republic of Korea

## Abstract

Conventional functional magnetic resonance imaging (fMRI) technique known as gradient-recalled echo (GRE) echo-planar imaging (EPI) is sensitive to image distortion and degradation caused by local magnetic field inhomogeneity at high magnetic fields. Non-EPI sequences such as spoiled gradient echo and balanced steady-state free precession (bSSFP) have been proposed as an alternative high-resolution fMRI technique; however, the temporal resolution of these sequences is lower than the typically used GRE-EPI fMRI. One potential approach to improve the temporal resolution is to use compressed sensing (CS). In this study, we tested the feasibility of *k-t* FOCUSS—one of the high performance CS algorithms for dynamic MRI—for non-EPI fMRI at 9.4T using the model of rat somatosensory stimulation. To optimize the performance of CS reconstruction, different sampling patterns and *k-t* FOCUSS variations were investigated. Experimental results show that an optimized *k-t* FOCUSS algorithm with acceleration by a factor of 4 works well for non-EPI fMRI at high field under various statistical criteria, which confirms that a combination of CS and a non-EPI sequence may be a good solution for high-resolution fMRI at high fields.

## 1. Introduction

Functional magnetic resonance imaging (fMRI) has had a wide impact in both the research and clinical community since its development. In conventional fMRI studies, positive blood oxygen level-dependent (BOLD) response signal is used as a measure to map neural activity in the brain [1], and the most common MR pulse sequence for acquiring BOLD fMRI images has been gradient-recalled echo (GRE) echo-planar imaging (EPI) due to its fast acquisition speed and high sensitivity to BOLD effect. However, this technique is susceptible to local magnetic field inhomogeneity and becomes sensitive to image distortion and degradation especially at high magnetic fields. Non-EPI sequences such as spoiled gradient echo or balanced steady-state free precession (bSSFP) can be used as an alternative tool for fMRI [2–9]; however, the major drawback of using these sequences for fMRI studies is the low temporal resolution compared to the typically used GRE-EPI.

One solution to overcome the low temporal resolution of non-EPI sequences is to adopt parallel imaging technique [10–12]. Although proven useful, the usage of parallel imaging results in reduced signal-to-noise ratio (SNR) due to the acceleration factor, the geometric factor of the different coil elements, and the *k*-space filling trajectory. The other solution to improve the temporal resolution of non-EPI sequences is to use compressed sensing (CS) [13–15]. CS theory states that it is possible to reconstruct an aliasing-free image even at sampling rates dramatically lower than the Nyquist sampling limit, as long as the nonzero signal is sparse and sampled incoherently. These requirements can be well satisfied in dynamic MRI, since arbitrary trajectories can be implemented to incoherently sample data and dynamic MR images can be sparsified due to high temporal redundancy [16, 17]. Recently, CS theory was successfully applied to dynamic MRI in a new algorithm called* k-t* FOCUSS by employing random sampling pattern in *k*-*t* space and by using various sparsifying temporal transforms such as Fourier transform (FT) and Karhunen-Loéve transform (KLT) to utilize the temporal redundancies [16, 18].

Though CS theory has gained attraction for its vast potential for MRI application, CS had been successfully applied to fMRI in only a few studies in the past. In most of the studies, CS was applied to GRE-EPI fMRI: ordinary GRE-EPI [19, 20] and spiral scan GRE-EPI [21]. Despite its application, GRE-EPI is generally known to suffer from the contribution of magnetic field inhomogeneity, which can degrade the performance of CS algorithms. Recently, it has been reported that application of CS to GRE-fMRI may increase statistical performance of activation detection [22]. Non-EPI sequences such as bSSFP or GRE may work better with CS algorithms than GRE-EPI, since the sequences utilize different RF excitations for each TR.

The signal dynamics of steady-state sequences such as bSSFP are known to be more complicated than other conventional sequences and may need careful examination prior to the application of CS to real data. Also, due to the large degree of freedom in CS application, it is important to understand the artifacts and effects related to CS reconstruction without any confounding factors from true physical artifacts. Thus, an extensive simulation study prior to actual CS application is required to reconstruct data appropriately, preserve fMRI signal details, and eventually create a general CS framework.

In this study, we tested the feasibility of CS for non-EPI fMRI at 9.4T using the model of rat somatosensory stimulation. Fully sampled data with high-resolution spoiled gradient echo and 4 independent pass-band bSSFP fMRI each with different phase-cycling angles (0°, 90°, 180°, and 270°) underwent retrospective downsampling and reconstruction using* k-t* FOCUSS algorithm. Various sampling patterns and sparsifying transforms such as temporal FT and KLT were employed to systematically study the effects of different *k*-space sampling pattern and the effects of choosing a different CS reconstruction algorithm in high field CS fMRI. The baseline image quality and sensitivity and specificity of activation maps from data with CS reconstruction were compared to those from the original full-sampled data. The potential for improving the temporal resolution of non-EPI fMRI at high magnetic fields without sacrificing quality of fMRI activation maps is demonstrated in this paper.

## 2. Methods

### 2.1. Animal Preparation and Data Acquisition

Three male Sprague-Dawley rats weighing 250~450 g (Charles River Laboratories, Wilmington, MA, USA) were used with the approval from the Institutional Animal Care and Use Committee (IACUC) at University of Pittsburgh. Animal preparation was the same as previously published [7]. Briefly, the rats were intubated for mechanical ventilation (RSP-1002, Kent Scientific, CT, USA). The catheters were inserted in the femoral artery and femoral vein for blood gas sampling and fluid administration (5% dextrose in saline infused at 0.4 mL/hr), respectively. Once the surgery was finished, the isoflurane level was maintained at 1.4%. Ventilation rate and volume were adjusted based on blood gas analysis results (Stat profile pHOx; Nova Biomedical, MA, USA).

Electrical stimulation was applied to either the right or left forepaw using two needle electrodes inserted under the skin between digits 2 and 4 [23]. Stimulation parameters for activation studies were as follows: current = 1.2~1.6 mA, pulse duration = 3 ms, repetition rate = 6 Hz, stimulation duration = 15 s, and interstimulation period = 3 min.

All experiments were carried out on a Varian 9.4T/31 cm MRI system (Palo Alto, CA) with an actively shielded gradient coil of 12 cm inner diameter, which operates at a maximum gradient strength of 40 G/cm and rise time of 120 *μ*s. A homogeneous coil and a surface coil (Nova Medical, Wilmington, MA) were used for RF excitation and reception, respectively. Localized shimming was performed with point resolved spectroscopy [24] over a coronal slab (~12 × 6 × 6 mm^3^) covering forelimb somatosensory cortex to yield a water spectral linewidth of 20~30 Hz. Spoiled gradient echo (which is denoted by GRE throughout this paper) and pass-band bSSFP studies were performed with TR/TE = 20/10 ms and 10/5 ms, respectively. The bSSFP fMRI studies were performed with four different phase-cycling angles (*θ*) of 0°, 90°, 180°, and 270° (which are denoted by PC 0, PC 1, PC 2, and PC 3, resp., throughout this paper). The resolution parameters were the same for all studies: matrix size = 256 × 192, FOV = 2.4 × 2.4 cm^2^, number of slice = 1, and slice thickness = 2 mm. Flip angles for all the bSSFP and GRE fMRI studies were 16° and 8°, respectively. Forty-eight measurements were acquired for each bSSFP fMRI study: 16 during prestimulus baseline, 8 during stimulation, and 24 during the poststimulus period. These numbers of measurements were reduced by half for GRE fMRI study, in order to maintain the same spatial resolution. Four bSSFP and one GRE fMRI studies composed one full set and each full set was repeated 15 to 25 times for averaging per subject rat.

### 2.2. **k**-Space Sampling Patterns

**Figure 1:**
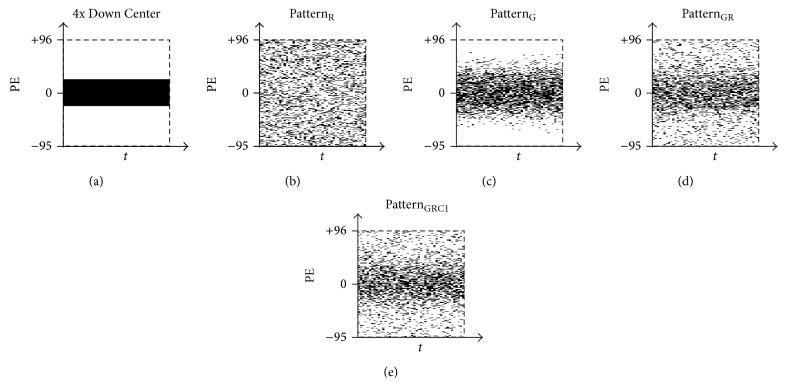
Masks of five different sampling patterns with downsampling factor of 4. Patterns of (a) full sampling of *k*-space center, (b) uniform random sampling, (c) Gaussian random sampling, (d) mixture of Gaussian and uniform random sampling, and (e) mixture of Gaussian and uniform random sampling with full sampling of center 1 line are shown. All masks are generated to sample only a quarter of the total data (total size of the data indicated by dashed box). Notice that no *k*-space high frequency information is included in (a) and (c).

The initial data to undergo CS reconstruction is important to guarantee high performance of CS algorithms. All fMRI studies in this work were conducted with block design paradigm; thus downsampling was considered in the *k*-*t* (i.e., *k*-space-temporal) domain to utilize CS algorithms optimized for exploiting temporal redundancy in dynamic MRI. In order to determine the optimal sampling pattern for CS application on fMRI data acquired at high field, four different sampling patterns were considered (Figures 1(b)–1(e)): sampling masks were generated using uniform random sampling (Figure 1(b)), Gaussian random sampling (Figure 1(c)), a mixture of Gaussian and uniform random sampling (Figure 1(d)), and a mixture of Gaussian and uniform random sampling with full sampling of *k*-space center 1 line (Figure 1(e)). The generated sampling masks were applied to each full-sampled *k*-space dataset for retrospective downsampling before the CS reconstruction procedure.

In this study, a fixed downsampling factor of 4 was applied to all datasets: only a quarter of the original *k*-space data was used for CS reconstruction. Each 2D sampling mask was generated for each time frame and a fixed number of *N*_PE_/4 lines were sampled along the phase-encoding (PE) direction to maintain the downsampling factor of 4 (where *N*_PE_ indicates the total number of PE lines). Note that bSSFP or GRE sequences utilize different RF excitations for each TR; thus the acceleration factor depends on the total number of PE lines sampled. The uniform random sampling mask was generated by sampling the PE lines according to a uniform probability distribution. The Gaussian random sampling mask was generated by sampling the PE lines according to a Gaussian probability distribution of *P*(*k*_*y*_) = *Ae* ^−(*k*_*y*_)^2^/2*σ*^2^^, with *σ* = *N*_PE_/9 (where *k*_*y*_ indicates *k*-space PE line number index in the range of −95 to 96, *σ* indicates standard deviation, and *A* indicates the weighting factor which was adjusted to make the equation a valid probability density function). The mixture of Gaussian and uniform random sampling mask was generated by sampling *N*_PE_/6 number of PE lines according to the above Gaussian probability distribution and subsequently sampling *N*_PE_/12 number of PE lines according to a uniform random probability distribution (*N*_PE_/6 + *N*_PE_/12 = *N*_PE_/4 lines were sampled in total). The mixture of Gaussian and uniform random sampling with full sampling of *k*-space center 1 line was generated similarly, but with continuous sampling of the *k*-space center 1 line (i.e., *k*_*y*_ = 0) for each time frame along the temporal dimension. Pseudocodes for generation of the sampling patterns are provided as follows.

*Pseudocode for Generation of k-Space Downsampling Pattern on 2D Time-Series MRI Data.*

For downsampling factor (*D*), one has the following.

(A) Uniform Random Sampling Mask
  (1) Generate uniform probability distribution along the PE dimension of *k*-space data.
  (2) Sample *N*_PE_/*D* number of PE lines according to the uniform probability distribution.
  (3) Repeat steps (1)-(2) for each time frame.
(B) Gaussian Random Sampling Mask
  (1) Define *σ* in relation to *N*_PE_.
  (2) Generate Gaussian probability distribution *P*(*k*_*y*_) = *Ae* ^−(*k*_*y*_)^2^/2*σ*^2^^ along the PE dimension of *k*-space data.
  (3) Sample *N*_PE_/*D* number of PE lines according to the Gaussian probability distribution.
  (4) Repeat steps (1)–(3) for each time frame.
(C) Mixture of Gaussian and Uniform Random Sampling Mask (Gaussian : Random = *a* : *b*)
  (1) Define *σ* in relation to *N*_PE_.
  (2) Generate Gaussian probability distribution *P*(*k*_*y*_) = *Ae* ^−(*k*_*y*_)^2^/2*σ*^2^^ along the PE dimension of *k*-space data.
  (3) Sample (*N*_PE_/*D*)×(*a*/(*a* + *b*)) number of PE lines according to the Gaussian probability distribution.
  (4) Generate uniform probability distribution along the PE dimension for the remaining PE lines.
  (5) Sample (*N*_PE_/*D*)×(*b*/(*a* + *b*)) number of PE lines according to the uniform probability distribution.
  (6) Repeat steps (1)–(5) for each time frame.
(D) Mixture of Gaussian and Uniform Random Sampling Mask with Full Sampling of Center 1 Line (Gaussian : Random = *a* : *b*)
  (1) Sample 1 PE line from the *k*-space center (e.g., *k*_*y*_ = 0).
  (2) Repeat all steps from (C) with *N*_PE_/*D* − 1 number of PE lines.

The Gaussian probability distribution was considered as a derivative form of *k*-space center-weighted downsampling pattern, with stronger weighting on *k*-space low-frequency information. Sampling using a mixture of Gaussian and uniform random probability distributions was considered as a further variation preserving information at both high and low frequencies. The inclusion of *k*-space center 1 line was considered as an option to preserve the lowest frequency information, since the center of *k*-space contains most of the contrast information for an MR image. For comparison analysis, original full-sampled data was used as the ground truth to study the effect of CS reconstruction, and a simple quarter downsampled mask with full sampling of *k*-space center (Figure 1(a)) was used as a control. Throughout the paper, uniform random sampling pattern, Gaussian random sampling pattern, mixture of Gaussian and uniform random sampling pattern, and mixture of Gaussian and uniform random sampling pattern with full sampling of center 1 line will be denoted by Pattern_R_, Pattern_G_, Pattern_GR_, and Pattern_GRC1_, respectively.

### 2.3. CS Algorithms

Two variations of* k-t* FOCUSS algorithms, temporal FT and KLT (which will be further denoted by Algorithms 1 and 2, resp.), were used in this study (see the Appendix for detailed description of* k-t* FOCUSS algorithms) [16]. The covariance matrix for Algorithm 2 was defined to be constructed from an initial reconstruction using Algorithm 1 with preliminary parameter of *N*_FOC1_ = 2 for each dataset:

(1) $$\begin{array}{c} C={\hat{U}}^{H}\hat{U}, \end{array}$$

where $\hat{U}=[{\hat{u}}_{1},{\hat{u}}_{2},\ldots ,{\hat{u}}_{T}]$ indicates the reconstruction from Algorithm 1 (please refer to the Appendix for detailed definition of $\hat{U}$). The eigenvectors of the covariance matrix were further used as the KL transform (Φ) and *N*_KLT_ = 1 was used to update Φ once.

### 2.4. *k-t* FOCUSS Parameters

For both algorithms, weighting matrix power factor (*p*) of 0.5 was used to find the sparse solution equivalent to the *l*_1_ solution of CS [16]. Conjugate gradient (CG) iteration number (*N*_CG_) of 30 was considered sufficient and was used for both Algorithm 1 (*N*_CG1_) and Algorithm 2 (*N*_CG2_), based on previous application with* k-t* FOCUSS [25]. Regularization factor (*λ*) of 0.1 was used for Algorithm 1 (*λ*_1_) [16] and 0.01 was used for Algorithm 2 (*λ*_2_) [22].

Both Algorithms 1 and 2 were optimized based on variation in the FOCUSS iteration number (*N*_FOC_) parameters (i.e., *N*_FOC1_ and *N*_FOC2_, resp.). The following stopping criterion is used to determine the optimal *N*_FOC_ value from each dataset in training phase:

(2) $$\begin{array}{c} \frac{{\left\|{\hat{U}}^{\left(k\right)}-{\hat{U}}^{\left(k-1\right)}\right\|}_{F}}{{\left\|{\hat{U}}^{\left(k\right)}\right\|}_{F}}<0.1, \end{array}$$

where ${\hat{U}}^{\left(k\right)}$ denotes the CS reconstruction of the spatiotemporal fMRI data at *k*th FOC iteration, ${\hat{U}}^{\left(k-1\right)}$ denotes the CS reconstruction of the spatiotemporal fMRI data at (*k* − 1)th FOC iteration, and ‖·‖_*F*_ denotes the Frobenius norm. The performance of* k-t* FOCUSS algorithms with the proposed stopping criterion for *N*_FOC_ parameters was evaluated via subject-based leave-one-out cross-validation (i.e., the *N*_FOC_ value for data from each subject was determined based on a training set consisting of data from the other subjects).

The effects of *N*_CG_ and *λ* were investigated separately for verification with the determined optimal *N*_FOC_ value (i.e., value found during the training phase) for each subject data. The reconstruction of phase-cycled bSSFP and GRE data was used for investigation with all cases of sampling patterns as follows. Residual error = ${\left\|y-\hat{y}\right\|}_{2}^{2}$ was used as a measure to observe data fitting and convergence in each* k-t* FOCUSS algorithm with increase in *N*_CG_, where **y** denotes the *k*-space time-series data of the sampled *k*-space lines and $\hat{y}$ denotes the CS reconstruction of the *k*-space time-series data for the corresponding *k*-space lines. Average mean square error (MSE) of the whole time-series data was used as a measure to observe the noise level in the reconstructed image with variation in *λ*, which was calculated as follows:

(3) $$\begin{array}{c} Average\;MSE=\frac{\sum_{t=1}^{T}{\left\|u_{t}-{\hat{u}}_{t}\right\|}_{2}^{2}}{TN}, \end{array}$$

where **u**_*t*_ denotes the original full-sampled spatiotemporal fMRI data at time frame *t*, ${\hat{u}}_{t}$ denotes the CS reconstructed spatiotemporal fMRI data at time frame *t*, *T* denotes the total number of time frames, and *N* denotes the total number of image pixels at each time frame.

### 2.5. Region of Interest Selection

A region of interest (ROI) was selected to help compare the effects of different sampling patterns and CS algorithms. The regions determined to be functionally active (i.e., rejecting the null hypothesis *H*_0_) according to the *t*-statistics map of the original full-sampled data were chosen as the ROI for further analysis. New ROIs were defined for each dataset.

### 2.6. Quantitative Analysis

Frame-by-frame normalized MSE, *t*-statistics functional map, ROI time course plot, and receiver operating characteristics (ROC) curve were calculated for further investigation of the applicability of CS for fMRI data at high field. These analyses were performed on the bSSFP data with PC2 for clear evaluation of the effect of CS application, since the sequence corresponds to the conventional bSSFP sequence (i.e., 180° phase-cycling) displaying a fairly uniform signal contrast without any significant banding artifacts and showed clear activation foci in the full-sampled data.

The frame-by-frame normalized MSE calculation at time *t* was performed using the following equation:

(4) $$\begin{array}{c} Frame\text{-}by\text{-}frame\;normalized\;MSE\left(t\right)=\frac{{\left\|u_{t}-{\hat{u}}_{t}\right\|}_{2}^{2}}{{\left\|u_{t}\right\|}_{2}^{2}}. \end{array}$$

Student's *t*-test was performed for each dataset to statistically analyze fMRI data and generate the *t*-statistics functional map. The *T*-score is calculated on a pixel by pixel basis over time as follows:

(5) $$\begin{array}{c} T\text{-}score=\frac{\bar{x}-\bar{y}}{\sqrt{s_{x}^{2}/n_{x}+s_{y}^{2}/n_{y}}}, \end{array}$$

where $\bar{\cdot }$ denote the mean, *s*_·_ denote the standard deviation, and *n*_·_ denote the length of the baseline time-series *x* and activation time-series *y*, respectively. The *t*-statistics functional map was generated for a significance level of 0.05, and clusters less than 6 pixels were rejected. ROI time course was plotted as the mean ROI value.

The ROC curve was generated to provide standardized and statistically meaningful means for comparing fMRI signal-detection accuracy [26]. For each dataset, the *t*-statistics map generated from the original full-sampled data with significance level of 0.05 was used as the ground truth. True positive fraction (TPF) and false positive fraction (FPF) were calculated over various significance levels to generate the ROC curve. The performance was measured by the area under the curve (AUC) ranging from 0 to 1, with 1 representing better performance. The TPF and FPF were calculated using the following equations:

(6) TPF=Number  of  True-Positive  Activation  Voxels×Number  of  Truly  Activated  Voxels  fromx3  x3Ground  Truth−1,FPF=(Number  of  False-Positive  Activation  Voxels)×Number  of  Truly  Non-Activated  Voxelsx3  x3from  Ground  Truth−1,

where TPF relates to sensitivity and 1 − FPF relates to specificity.

## 3. Results

### 3.1. Determination of **N**_FOC_

*N*_FOC_ values determined via subject-based leave-one-out cross-validation for Algorithm 1 were 4, 3, 3, and 3 for Pattern_R_, Pattern_G_, Pattern_GR_, and Pattern_GRC1_, respectively, and those for Algorithm 2 were 5, 4, 4, and 4 for Pattern_R_, Pattern_G_, Pattern_GR_, and Pattern_GRC1_, respectively. Identical *N*_FOC_ values were found regardless of the pulse sequence type (i.e., GRE and bSSFP PC0, PC1, PC2, and PC3) in all the subjects. All further analyses were performed with the determined *N*_FOC_ values for each subject data, to evaluate the performance of *k*-*t* FOCUSS algorithms with the proposed stopping criterion.

### 3.2. Original Data: High Field bSSFP

**Figure 2:**
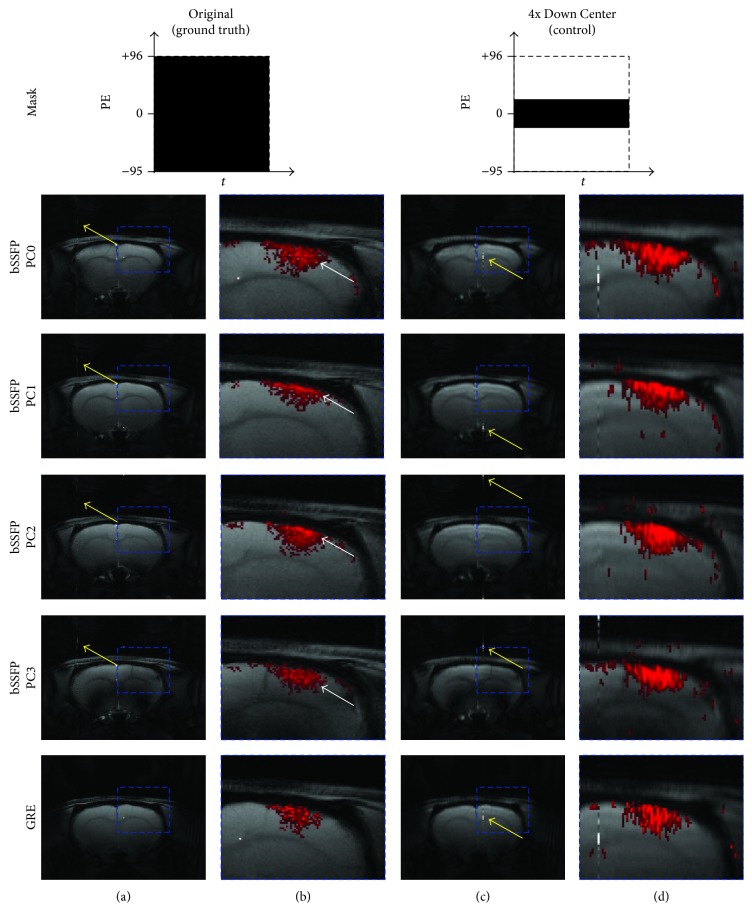
Baseline and fMRI images of original and control. (a) Baseline images and (b) fMRI maps of full-sampled data and (c) baseline images and (d) fMRI maps of downsampled data with full acquisition of *k*-space center are shown. The 20th and 10th time frame of bSSFP and GRE is shown for baseline images, respectively. The fMRI maps are shown for significance level of 0.05. Downsampling pattern and acquisition method are shown on the top and left-hand side of the images, respectively. The DC artifact is indicated by yellow arrow and the enlarged fMRI activation region is indicated by blue dashed box. Notice the activation foci shift (white arrow) in the phase-cycled bSSFP data. The case from a representative rat is shown since similar results were obtained for different subject rats.

Different phase-cycling angles in the fMRI maps of full-sampled bSSFP data showed shifting in activation foci (i.e., the activation foci were located around the cortical surface area for PC1 and 2, while they were located in the middle cortical regions for PC0 and 3, as indicated by white arrows in Figure 2(b)). This spatial shift of activation foci as a function of PC angle implies that the high field phase-cycled bSSFP maps are spatially heterogeneous due to magnetic field inhomogeneity and was used in this study to confirm that CS with an appropriate downsampling scheme can preserve the details of the spatial pattern of the functional activation.

### 3.3. Reconstruction with Algorithm 1:* k-t* FOCUSS with Temporal FT

**Figure 3:**
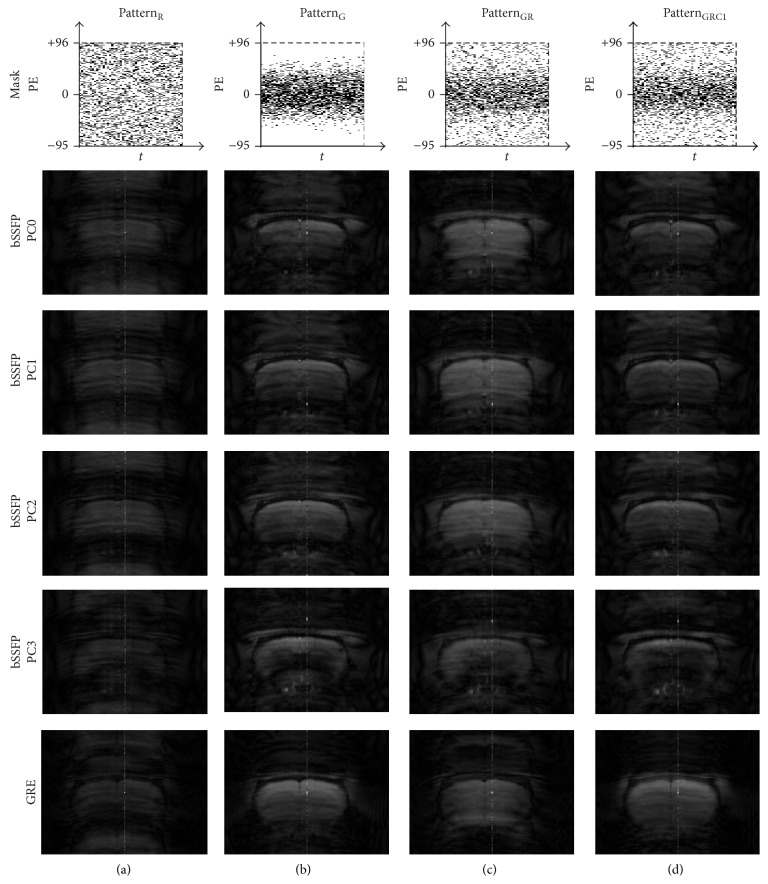
Baseline images after downsampling. Baseline images of downsampled data using (a) Pattern_R_, (b) Pattern_G_, (c) Pattern_GR_, and (d) Pattern_GRC1_ are shown. The 20th and 10th time frame of bSSFP and GRE is shown, respectively. Downsampling pattern and acquisition method are shown on the top and left-hand side of the images, respectively. Notice the blurring and artifacts in the downsampled images. The case from a representative rat is shown since similar results were obtained for different subject rats.

**Figure 4:**
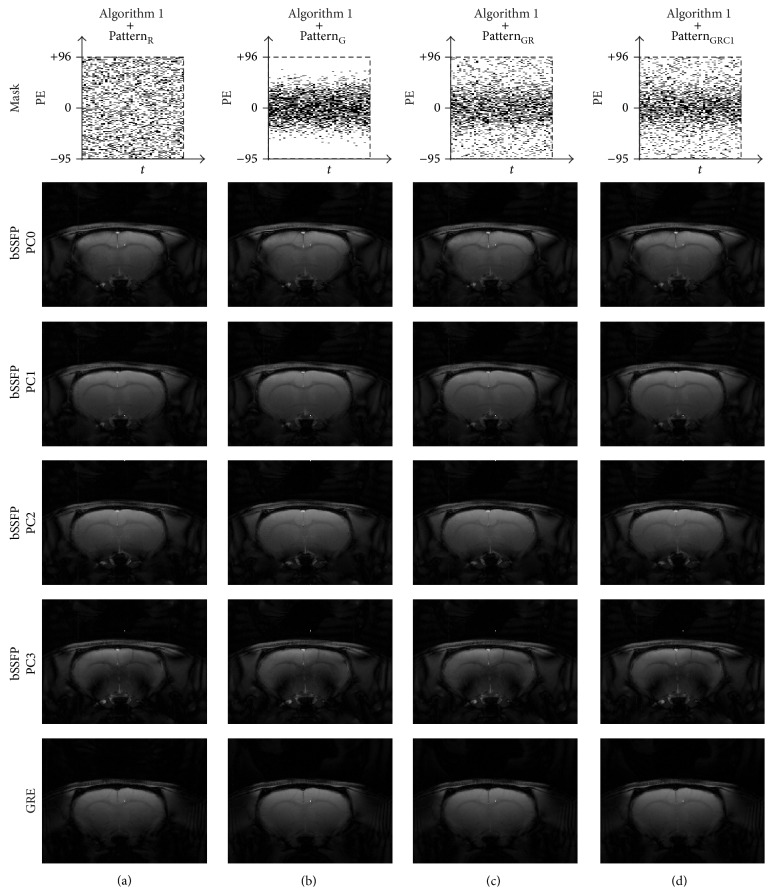
Comparison of baseline images reconstructed using Algorithm 1 (*k-t* FOCUSS with temporal FT). Baseline images of CS reconstructed data using Algorithm 1 and (a) Pattern_R_, (b) Pattern_G_, (c) Pattern_GR_, and (d) Pattern_GRC1_ are shown. The 20th and 10th time frame of bSSFP and GRE is shown, respectively. Downsampling pattern and acquisition method are shown on the top and left-hand side of the images, respectively. The case from a representative rat is shown since similar results were obtained for different subject rats.

**Figure 5:**
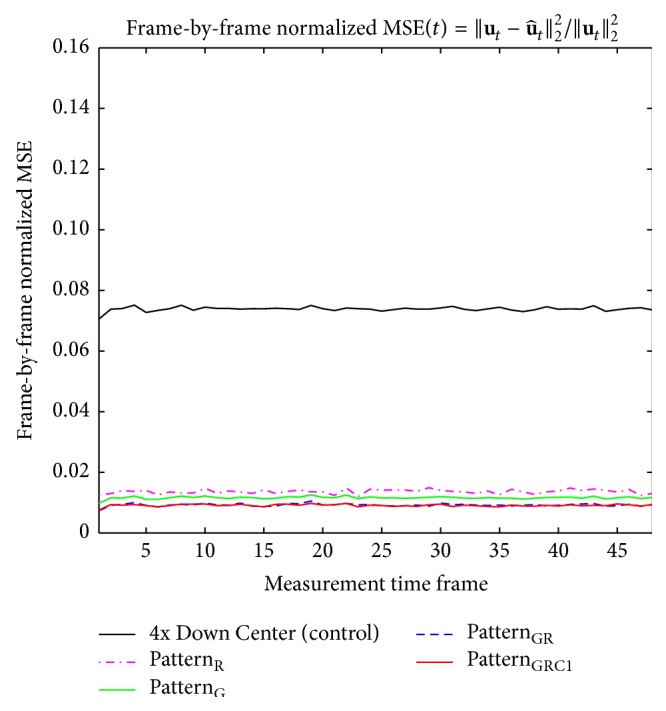
Comparison of frame-by-frame normalized MSE plots from reconstructed baseline images using Algorithm 1 (*k-t* FOCUSS with temporal FT). The frame-by-frame normalized MSE plots of bSSFP time-series data with PC angle of 180° are shown. The case from a representative rat is shown since similar results were obtained for different subject rats.

Visually the original baseline images became blurred with artifacts after downsampling was applied (Figure 3). Despite the distortion and degradation after downsampling, the baseline images were well reconstructed using Algorithm 1 regardless of sampling pattern (Figures 4(a)–4(d)). Visually the image contrast and resolution were well preserved for CS reconstructed images from all sampling patterns compared to the original baseline image (Figure 2(a)) and downsampled baseline image with only *k*-space low-frequency information (Figure 2(c)). The frame-by-frame normalized MSE values from all the CS sampling patterns were significantly lower than those from downsampling with only *k*-space low-frequency information (Figure 5), indicating high reconstruction performance of Algorithm 1. In particular, the mixture of Gaussian and uniform random sampling scheme (i.e., Pattern_GR_ and Pattern_GRC1_) showed the lowest frame-by-frame normalized MSE values across all time frames. Overall, all Gaussian-weighted sampling patterns showed increased spatial resolution and SNR (Figures 4(b), 4(c), and 4(d)) with reduction of artifacts (indicated by yellow arrow in Figure 2(a)) for all phase-cycled bSSFP data, while downsampling with only *k*-space low-frequency information showed increase of artifacts (Figure 2(c)).

**Figure 6:**
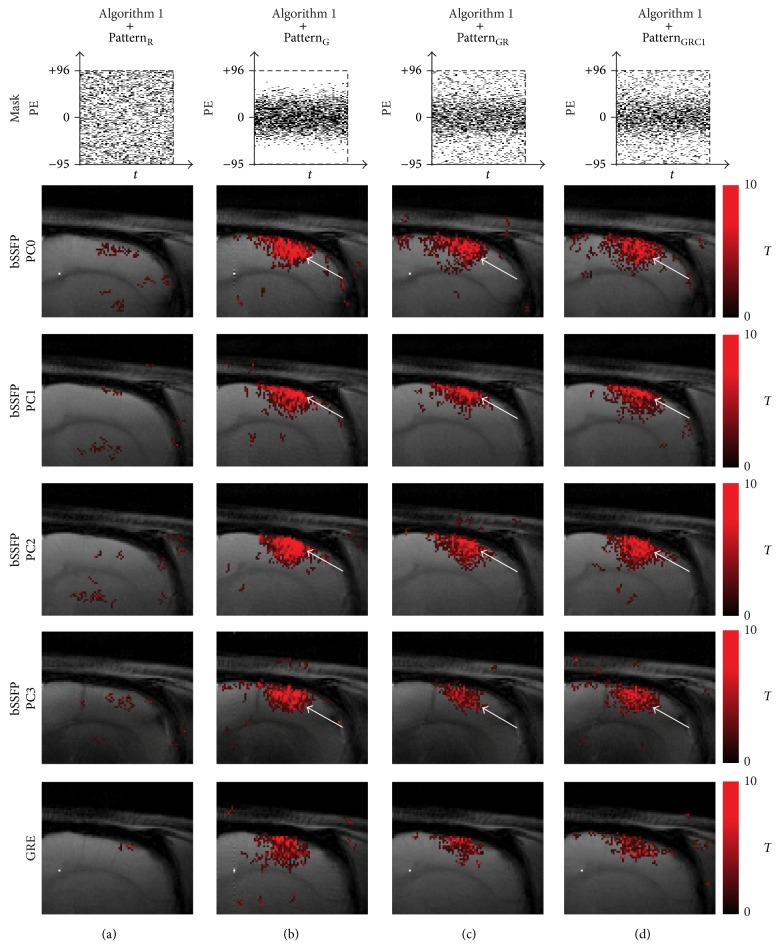
Comparison of fMRI maps reconstructed using Algorithm 1 (*k-t* FOCUSS with temporal FT). The fMRI maps of CS reconstructed data using Algorithm 1 and (a) Pattern_R_, (b) Pattern_G_, (c) Pattern_GR_, and (d) Pattern_GRC1_ are shown for significance level of 0.05. Downsampling pattern and acquisition method are shown on the top and left-hand side of the images, respectively. Notice the activation foci shift (white arrow) in the phase-cycled bSSFP data. The case from a representative rat is shown since similar results were obtained for different subject rats.

The fMRI maps were also reconstructed well from all Gaussian-weighted sampling patterns using Algorithm 1 (Figures 6(b), 6(c), and 6(d)), while those from Pattern_R_ did not show any meaningful functional activations (Figure 6(a)). The fMRI maps from downsampling with only *k*-space low-frequency information showed significant blurring in the activation region (Figure 2(d)). The fMRI maps from Pattern_GR_ (Figure 6(c)) and Pattern_GRC1_ (Figure 6(d)) were closer to the original fMRI maps than those from Pattern_G_ (Figure 6(b)) in terms of preserving details in activation foci shift, presumably due to the inclusion of appropriate high frequency *k*-space information.

**Figure 7:**
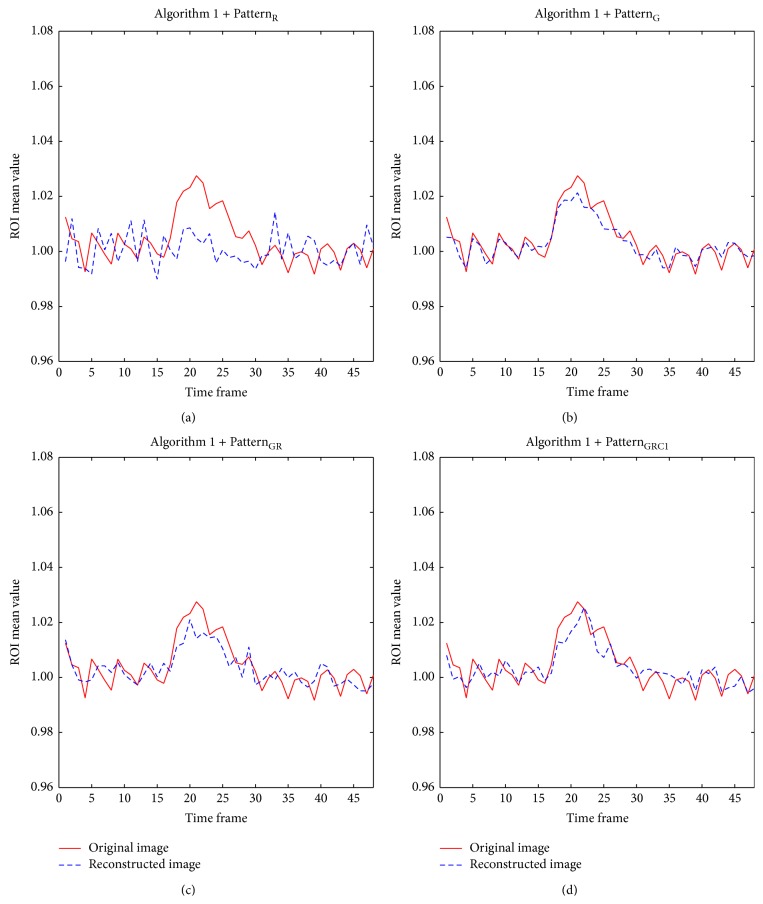
Comparison of mean ROI time course plots between full-sampled original data and CS reconstructed data using Algorithm 1 (*k-t* FOCUSS with temporal FT). The time course of mean ROI from CS reconstructed data using Algorithm 1 and (a) Pattern_R_, (b) Pattern_G_, (c) Pattern_GR_, and (d) Pattern_GRC1_ is shown. Mean ROI is calculated from bSSFP time-series data with PC angle of 180°. The case from a representative rat is shown since similar results were obtained for different subject rats.

The time course of the mean ROI value was also relatively well preserved in images from all Gaussian-weighted sampling patterns (Figures 7(b), 7(c), and 7(d)), while those from Pattern_R_ differed from the original with significantly increased temporal fluctuation (Figure 7(a)). Mean ROI time courses of images from all Gaussian-weighted sampling patterns resembled those of the original data; even with only (1/4)th of the whole data, the mean ROI time course followed the trend of the original time course with slightly reduced mean amplitude difference, percent signal change, and also signal fluctuation. These observations were applicable regardless of acquisition method and different PC angles for bSSFP. The AUC value of ROC curves indicated overall high sensitivity and specificity of all Gaussian-weighted sampling patterns using Algorithm 1 (Table 1). Pattern_GRC1_ displayed the highest ROC performance than other sampling patterns including downsampling with only *k*-space low-frequency information.

The reconstruction times of the* k-t* FOCUSS algorithms are shown for bSSFP PC2 and GRE in Table 2. Only one representative case is shown for each bSSFP and GRE data since similar results were obtained regardless of bSSFP PC angle, sampling pattern, and subject rats, despite the difference in total time due to the usage of different *N*_FOC_ parameter values. The reconstruction time for the bSSFP data was approximately twice as long as that of the GRE data, since the speed of reconstruction is largely dependent on the size of the matrix and number of time frames (recall that the number of time frames of GRE data was half of that of bSSFP data).

### 3.4. Reconstruction with Algorithm 2:* k-t* FOCUSS with KLT

**Figure 8:**
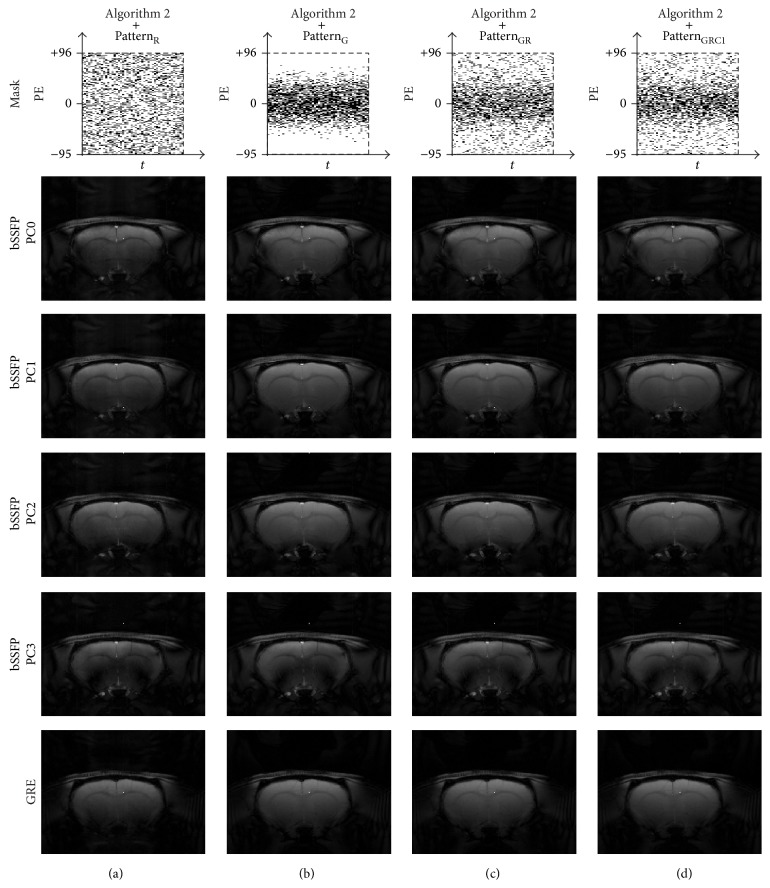
Comparison of baseline images reconstructed using Algorithm 2 (*k-t* FOCUSS with KLT). Baseline images of CS reconstructed data using Algorithm 2 and (a) Pattern_R_, (b) Pattern_G_, (c) Pattern_GR_, and (d) Pattern_GRC1_ are shown. The 20th and 10th time frame of bSSFP and GRE is shown, respectively. Downsampling pattern and acquisition method are shown on the top and left-hand side of the images, respectively. The case from a representative rat is shown since similar results were obtained for different subject rats.

**Figure 9:**
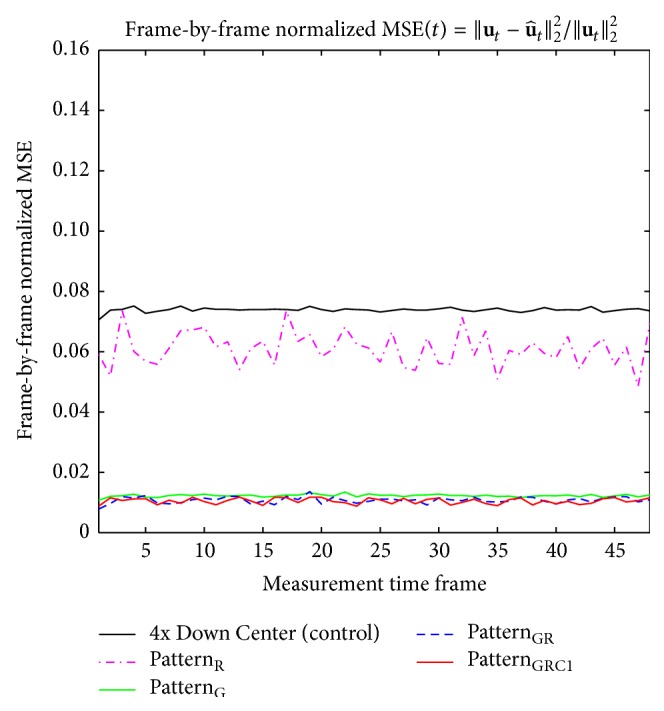
Comparison of frame-by-frame normalized MSE plots from reconstructed baseline images using Algorithm 2 (*k-t* FOCUSS with KLT). The frame-by-frame normalized MSE plots of bSSFP time-series data with PC angle of 180° are shown. The case from a representative rat is shown since similar results were obtained for different subject rats.

**Figure 10:**
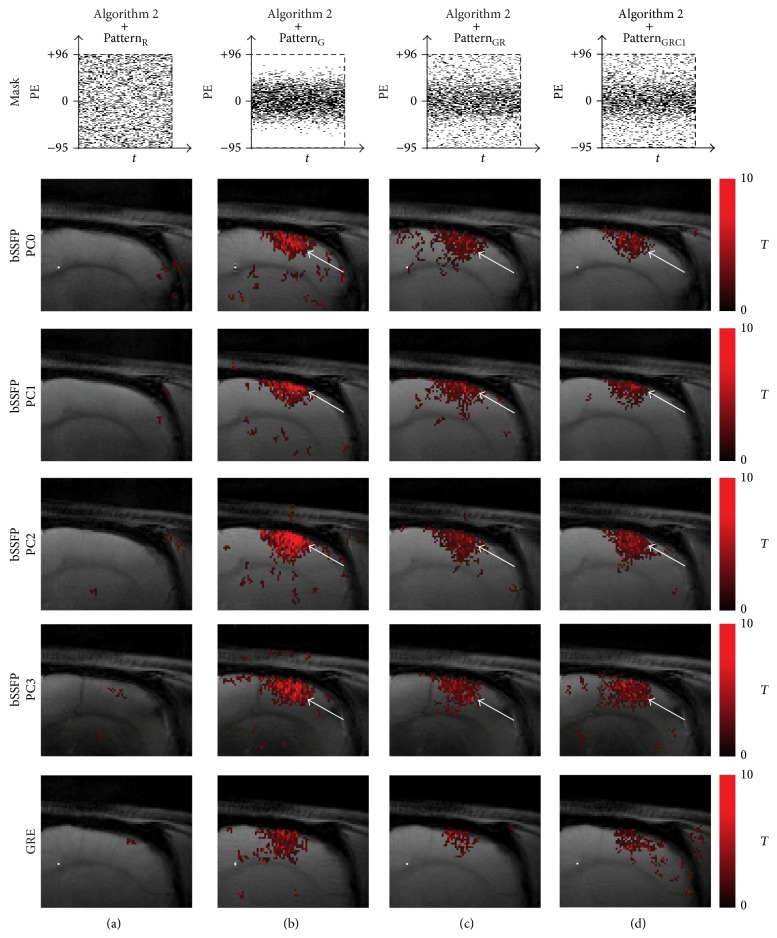
Comparison of fMRI maps reconstructed using Algorithm 2 (*k-t* FOCUSS with KLT). fMRI maps of CS reconstructed data using Algorithm 2 and (a) Pattern_R_, (b) Pattern_G_, (c) Pattern_GR_, and (d) Pattern_GRC1_ are shown for significance level of 0.05. Downsampling pattern and acquisition method used are shown on the top and left-hand side of the images, respectively. Notice the activation foci shift (white arrow) in the phase-cycled bSSFP data. The case from a representative rat is shown since similar results were obtained for different subject rats.

**Figure 11:**
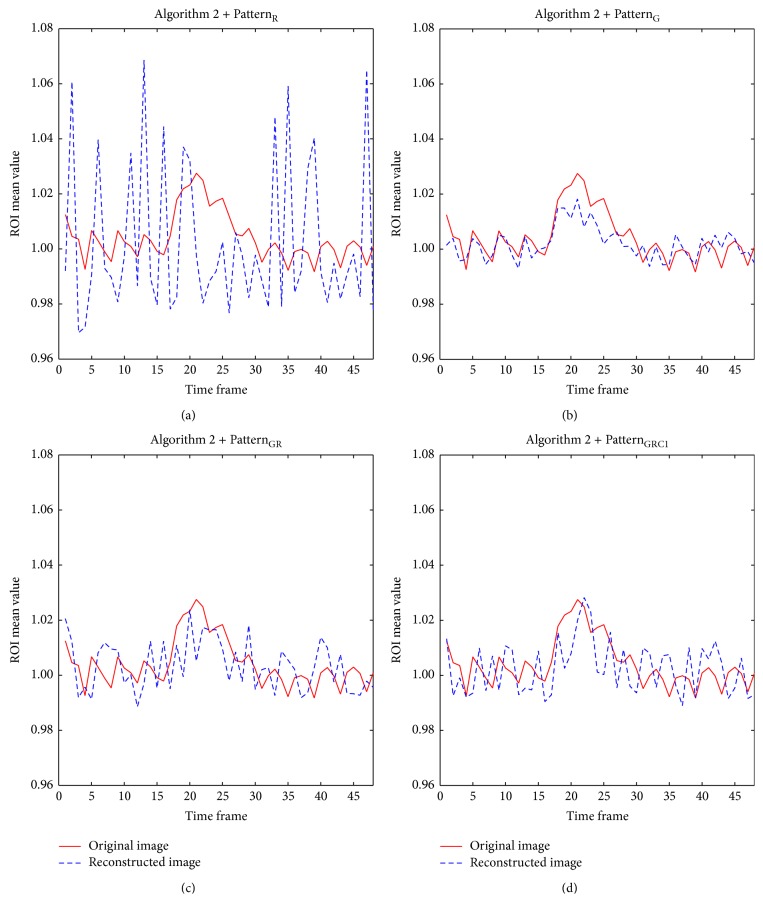
Comparison of mean ROI time course plots between full-sampled original data and CS reconstructed data using Algorithm 2 (*k-t* FOCUSS with KLT). The time course of mean ROI from CS reconstructed data using Algorithm 2 and (a) Pattern_R_, (b) Pattern_G_, (c) Pattern_GR_, and (d) Pattern_GRC1_ is shown. Mean ROI is calculated from bSSFP time-series data with PC angle of 180°. The case from a representative rat is shown since similar results were obtained for different subject rats.

Results of Algorithm 2 in terms of baseline images, frame-by-frame normalized MSE plot, fMRI maps, ROI time course, and AUC values in ROC curve are shown in Figures 8, 9, 10, and 11 and Table 1, respectively. Overall the results were similar to those of Algorithm 1. Slight differences were observed between algorithms in the view points of sensitivity and specificity depending on sampling pattern. The images from all sampling patterns for Algorithm 2 showed slightly lower sensitivity and specificity than those for Algorithm 1. The reconstruction time of Algorithm 2 was longer than that of Algorithm 1, mainly due to the calculation of covariance matrix requiring the preliminary estimation (Table 2).

### 3.5. Investigation of **N**_CG_ and ***λ*** Effect

**Figure 12:**
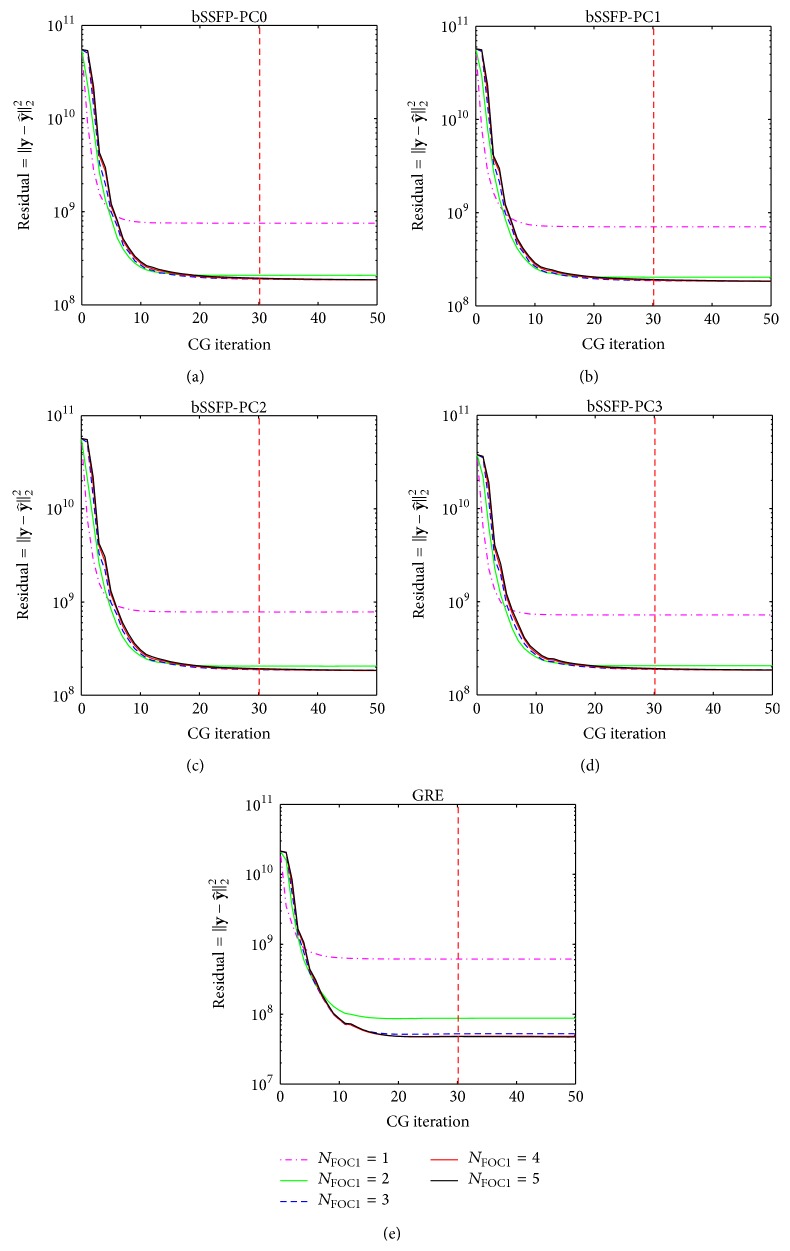
Example showing the effect of *N*_CG1_ on reconstruction using Algorithm 1 (*k-t* FOCUSS with temporal FT). Residual error with *N*_CG1_ variation using Algorithm 1 on (a) bSSFP PC0, (b) bSSFP PC1, (c) bSSFP PC2, (d) bSSFP PC3, and (e) GRE data is shown for each different *N*_FOC1_. Plots are obtained from reconstruction of downsampled data using Pattern_GRC1_ during testing phase of a representative rat, and similar results were obtained from different sampling patterns and different subject rats. Notice that residual error reaches convergence as *N*_CG1_ increases. The chosen *N*_CG1_ value of 30 is represented in each plot as a red dashed line.

**Figure 13:**
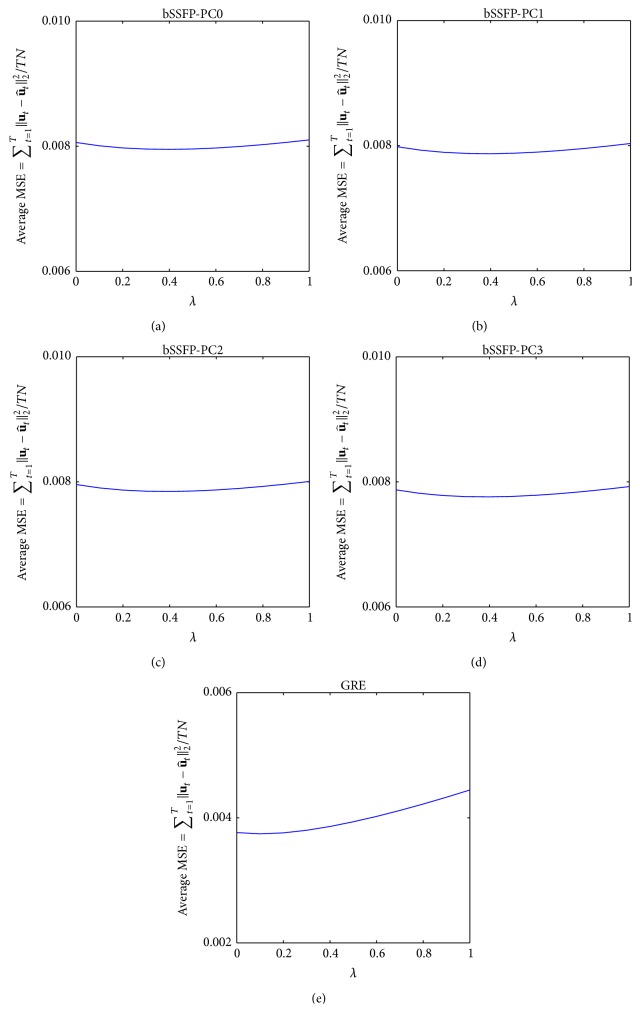
Example showing the effect of *λ*_1_ on reconstruction using Algorithm 1 (*k-t* FOCUSS with temporal FT). Average MSE with *λ*_1_ variation using Algorithm 1 on (a) bSSFP PC0, (b) bSSFP PC1, (c) bSSFP PC2, (d) bSSFP PC3, and (e) GRE data is shown. Plots are obtained from reconstruction of downsampled data using Pattern_GRC1_ and optimal *N*_FOC1_ found during testing phase of a representative rat, and similar results were obtained from different sampling patterns and different subject rats. Notice that reconstruction error is relatively invariant to *λ*_1_ variation and minimal error is achieved with small values of *λ*_1_.

**Figure 14:**
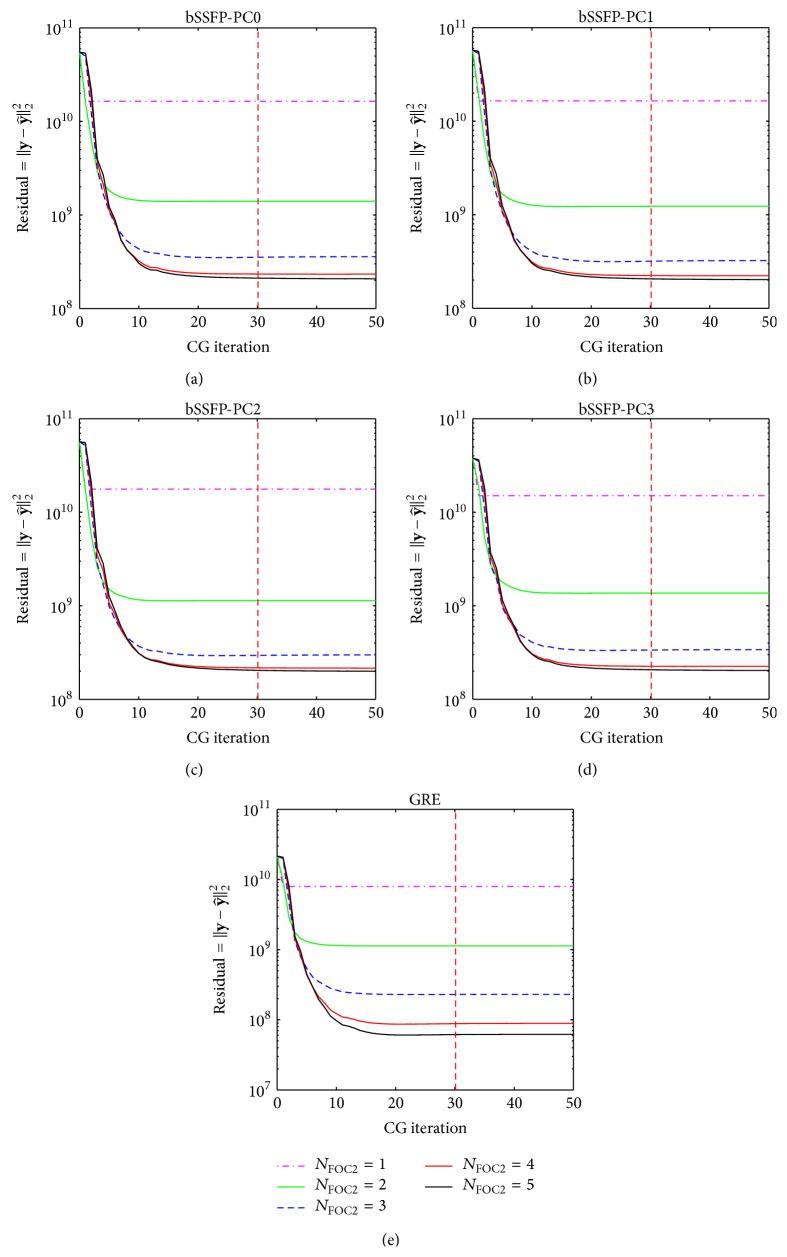
Example showing the effect of *N*_CG2_ on reconstruction using Algorithm 2 (*k-t* FOCUSS with KLT). Residual error with *N*_CG2_ variation using Algorithm 2 on (a) bSSFP PC0, (b) bSSFP PC1, (c) bSSFP PC2, (d) bSSFP PC3, and (e) GRE data is shown for each different *N*_FOC2_. Plots are obtained from reconstruction of downsampled data using Pattern_GRC1_ during testing phase of a representative rat, and similar results were obtained from different sampling patterns and different subject rats. Notice that residual error reaches convergence as *N*_CG2_ increases. The chosen *N*_CG2_ value of 30 is represented in each plot as a red dashed line.

**Figure 15:**
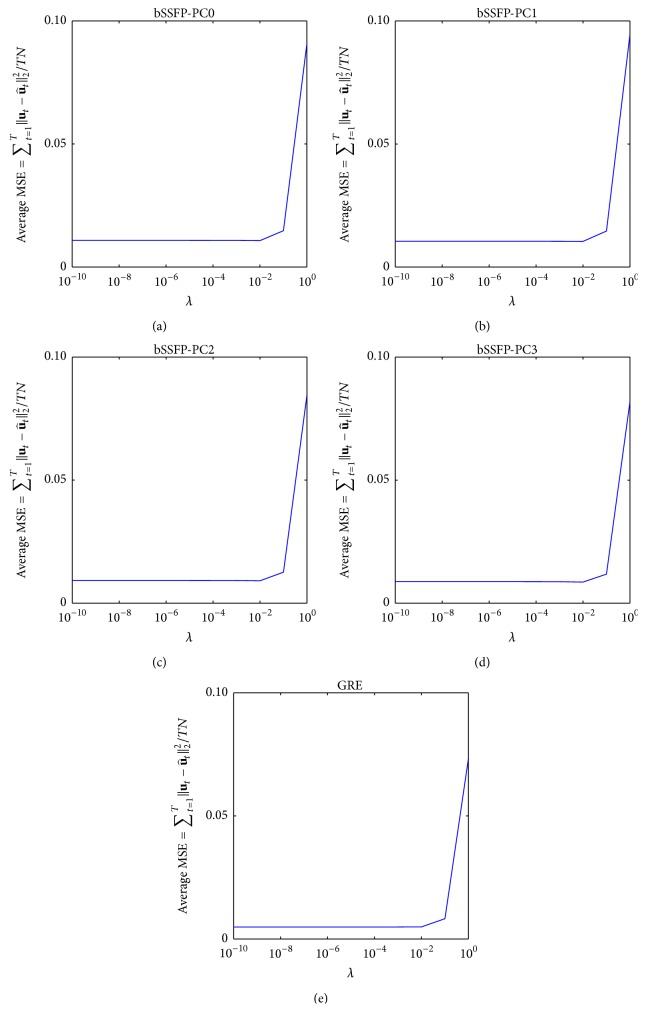
Example showing the effect of *λ*_2_ on reconstruction using Algorithm 2 (*k-t* FOCUSS with KLT). Average MSE with *λ*_2_ variation using Algorithm 2 on (a) bSSFP PC0, (b) bSSFP PC1, (c) bSSFP PC2, (d) bSSFP PC3, and (e) GRE data is shown. Plots are obtained from reconstruction of downsampled data using Pattern_GRC1_ and optimal *N*_FOC2_ found during testing phase of a representative rat, and similar results were obtained from different sampling patterns and different subject rats. Notice that reconstruction error is relatively invariant to *λ*_2_ variation and minimal error is achieved with small values of *λ*_2_.

Representative results from CS reconstruction of Pattern_GRC1_ using Algorithms 1 and 2 are shown in Figures 12, 13, 14, and 15, and similar results were obtained regardless of acquisition method and sampling patterns. Based on the results from Figures 12 and 14, the *N*_CG_ value of 30 (i.e., value used for both Algorithms 1 and 2 throughout the paper) seems to be sufficient to ensure data fitting and error convergence. As shown in Figures 13 and 15, the reconstruction error of* k-t* FOCUSS algorithms was relatively insensitive to *λ* variation and minimal error was achieved with small values of *λ* (e.g., less than 1).

## 4. Discussion

To our knowledge, it is the first study to apply CS to high field bSSFP fMRI and to systematically evaluate effects of CS sparsity schemes on non-EPI fMRI. The CS sampling scheme should be determined in relation to the CS algorithm to preserve detailed image information appropriately. Dense sampling in *k*-space center region such as Gaussian-weighting or inclusion of *k*-space center 1 line was better for CS, due to the fact that most energy is located in the *k*-space center region and also due to incoherent aliasing effects from variable-density sampling [13, 16]. The reconstruction results from Pattern_GR_ and Pattern_GRC1_ also verify that a variation in sampling scheme with more *k*-space high frequency information leads to better reconstruction performance preserving signal details (e.g., activation foci shifting phenomenon in bSSFP), which may become critical for applications such as fMRI studies. Inclusion of more *k*-space low-frequency information implies less *k*-space high frequency information which may lead to an enlargement or blurring of the activation foci in fMRI maps (e.g., Figures 2(d), 6(b), and 10(b)). Thus, both *k*-space center and edge regions are important, and methods that achieve a certain balance between them need to be exploited for correct reconstruction of non-EPI fMRI data using CS. Overall the mixture of Gaussian and uniform random sampling scheme reconstructed both the baseline images and fMRI maps well while preserving the signal details and thus seems to be an ideal sampling scheme for CS applied to non-EPI fMRI.

The two algorithms of* k-t* FOCUSS with temporal FT and KLT showed similar performances overall. The slight differences in their results are presumed to be due to the utilization of different transformation domains for each iteration. Interestingly,* k-t* FOCUSS with temporal FT performed slightly better than* k-t* FOCUSS with KLT in terms of ROC performance in this study, despite the fact that KLT is known as an efficient spectral decorrelator [27, 28]. Several factors may account for this. First, the fMRI studies were performed with block design paradigm in this work, and temporal redundancy from the spatial-temporal frequency domain may have been exploited better for such data type. Since KLT is a data-driven transform,* k-t* FOCUSS with KLT may potentially perform better in cases of rapid event-related paradigms. Second, the decorrelation of nonperiodic noise might not have been noticeable in the image, since bSSFP sequence is known to provide the highest SNR per unit time [29, 30] and the simulation studies were performed on datasets with enough averaging (e.g., 15 to 25 times). Recently, it has been reported that application of CS to fMRI can increase FPF in real acquisition settings, and* k-t* FOCUSS with KLT has shown to reconstruct fMRI maps with reduced false activations [22]. Thus, the effectiveness of both algorithms needs verification with real fMRI studies. Nonetheless, results from the current study indicate that both algorithms are potentially good solutions for acceleration of high field non-EPI fMRI.

Appropriate choice of CS reconstruction parameters is one of the main concerns of the application of CS. The optimal parameters may vary depending on noise level, temporal resolution, and other possible factors in actual data acquisition environment. In general, reconstruction parameters are found with known noise level [31] or alternatively are selected via cross-validation [32–34]. There are multiple parameters involved for the case of* k-t* FOCUSS algorithm, which requires hyperparameter optimization and thus increases the computation burden [16]. Therefore, the effect of two different* k-t* FOCUSS parameters, *λ* and *N*_CG_, is additionally investigated in this study. Considering the physical meaning of each parameter, two different metrics were used for evaluation. Since the regularization parameter *λ* is a tuning parameter used to find the solution with best improvement in SNR, average MSE was used to show its effect on the noise level in the reconstructed image. Since the CG method is employed to iteratively find the solution to the unknown signal (i.e., denoted by **x** in (A.9) of the Appendix), residual error was used to investigate data fitting and convergence with decreases in measurement error (i.e., difference between sampled *k*-space measurements **y** and estimation $\hat{y}$) as number of CG iterations increases (note that the signal-measurement relationship is defined in (A.4) of the Appendix and can be used to find $\hat{y}$ from **x**). Based on the results from Figures 12 and 14, fixation of *N*_CG_ to a sufficient value (e.g., 30 in case of our study) and *λ* to a small value is preferred to reduce parameter variability and to simplify the usage of* k-t* FOCUSS algorithms on high field non-EPI fMRI studies. These results agree with previous applications of* k-t* FOCUSS where a sufficient value of *N*_CG_ and a small value of *λ* are used and are proven to perform well in high-quality fMRI studies from real scanner acquisitions [16, 22]. However, care should be taken for extrapolation of these parameters for data types different from those of the current studies. With fixation of *N*_CG_ and *λ*, the only issue for the application of* k-t* FOCUSS algorithms for high field bSSFP fMRI data lies in the choice of *N*_FOC_. The *N*_FOC_ parameters found from the current study need to be tested in the context of real CS application for verification and general usage. The choice of a high *N*_FOC_ value may ensure minimal error for most cases of applications; however, this also leads to increased number of calculations required for CS reconstruction. Thus, the trade-off between minimization of error and increase in postprocessing time must be considered appropriately before choosing the *N*_FOC_ value for future studies.

**Figure 16:**
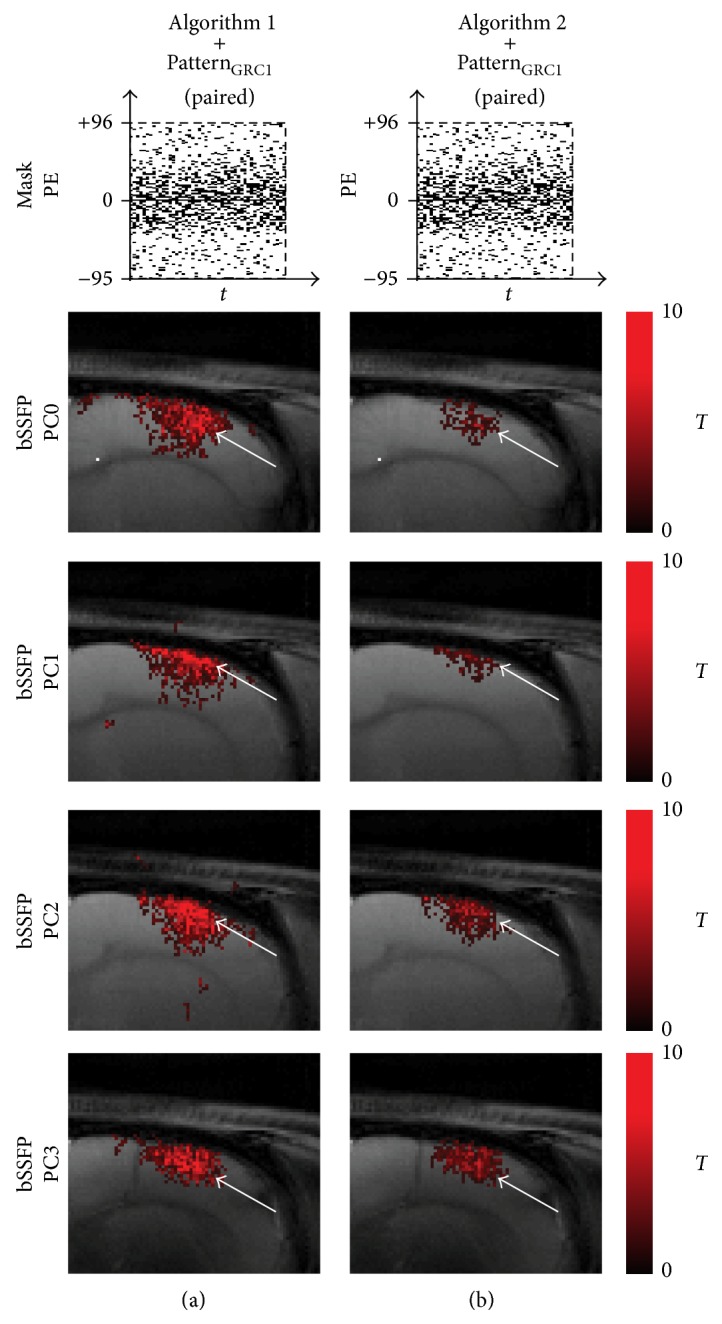
Presumed effect of paired random sampling scheme in the reconstruction of fMRI map using* k-t* FOCUSS algorithms. The fMRI maps of CS reconstructed data using (a) Algorithm 1 and pairwise sampling of Pattern_GRC1_ and (b) Algorithm 2 and pairwise sampling of Pattern_GRC1_ are shown for significance level of 0.05. Downsampling pattern and acquisition method used are shown on the top and left-hand side of the images, respectively. Notice the decrease in activation sensitivity with the inclusion of pairwise sampling scheme.

Eddy currents can cause problems in bSSFP imaging with nonlinear phase-encoding orders. The problems may become noticeable when the sparsity schemes tested in this study are implemented in real acquisition settings. Previously Bieri et al. [35] discovered a simple method to suppress the eddy current effect by pairing two consecutive *k*-space lines. By incorporating this idea, a simulation study was performed to see the effect of pairwise downsampling scheme. A paired Pattern_GRC1_ was generated with a downsampling factor of 4. The downsampling scheme and reconstructed fMRI maps from each* k-t* FOCUSS algorithm are shown in Figure 16, and the ROC performance of the reconstructed data is shown in Table 3. The employment of pairwise sampling scheme showed maintenance of activation foci shift but decrease in both activation detection sensitivity and specificity compared to the results without pairwise sampling (Figures 6(d) and 10(d)), regardless of PC angle and* k-t* FOCUSS algorithm. Overall, these results indicate that the pairwise sampling scheme may be used to suppress eddy current artifacts in bSSFP fMRI with CS, but there exists trade-off between the suppression of eddy current effect and fMRI sensitivity as well as specificity.

Application of CS to high field non-EPI fMRI can be meaningful for high-resolution fMRI studies, since conventional GRE-EPI fMRI is sensitive to image distortion and degradation caused by local magnetic field inhomogeneity at high magnetic fields. Although the temporal resolution of non-EPI sequences is lower than the typically used GRE-EPI, it is shown through the study that the temporal resolution or the spatial coverage can be improved using CS. Several potential advantages of CS can be derived for fMRI studies in this regard. First, better temporal resolution increases the number of time frames within a given time and can in turn improve the statistical power of BOLD activations [22, 36]. Second, the weighted-norm process of the CS algorithm can reduce artifacts from scanner-related drifts, respiratory-induced noise, cardiac pulsation, and subject motion [37–39] and can also improve the activation detection sensitivity in *t*-statistics [22]. Lastly, CS can improve spatial coverage which is essential for many fMRI studies that require a big ROI or ROIs from multiple brain regions. Thus, the application of CS in fMRI has great potential in practice.

One negative aspect of CS in fMRI studies is the addition of postprocessing time related to CS reconstruction. The reconstruction time of* k-t* FOCUSS algorithms is largely affected by the iteration parameters and the size of the data (e.g., matrix size, number of slices, number of time frames, etc.). The results from Table 2 imply that the temporal resolution or spatial coverage of non-EPI sequence fMRI studies can be improved using CS at the cost of reasonable addition of postprocessing time (i.e., several minutes). Therefore, depending on applications, the trade-off between reconstruction time and temporal resolution (and/or spatial coverage) must be investigated before applying CS algorithms to fMRI studies.

In the past, there have been many dynamic MRI studies other than fMRI with acceleration factor of 8 or higher using CS [18, 40–42]. However, CS has been applied to fMRI in a limited number of studies and acceleration factor up to 4 was used in most of the truly accelerated fMRI studies [21, 22]. Decrease in image quality has also been reported in some pilot studies after CS reconstruction even with 2-fold acceleration [21]. This may be attributed to the fact that distinct from other dynamic MRI studies fMRI requires detection of fine signal changes, which can be achieved by preserving high frequency information. Based on the results from our studies, acceleration factor of 4 seems sufficient for CS application on high field non-EPI fMRI studies. For example, for a bSSFP fMRI experiment with matrix size = 128 × 128 and TR = 5 ms, the temporal resolution becomes 0.64 s for a single slice. Thus, the 4-fold acceleration can improve the temporal resolution up to 0.16 s (i.e., close to the temporal resolution of EPI) or the spatial coverage up to 24 slices (i.e., near whole brain coverage) with temporal resolution less than 4 s (note that although TR was 10 ms in this study, bSSFP with TR ≤ 10 ms has been successfully applied to fMRI at high field ≥7T). Nonetheless, improvements can be made to increase the acceleration factor above 4 and the quality may depend on the scan condition (e.g., scan resolution, SNR). A systematic study for different acceleration factors may need to be conducted to further improve application of CS on high field non-EPI fMRI studies.

In this paper, the effect of CS is investigated through retrospective downsampling of full-sampled fMRI data. Therefore, further works are necessary to verify the downsampling schemes that were retrospectively evaluated in this study, by implementing them and performing real fMRI studies, which is beyond the scope of this paper.

## 5. Conclusion

The CS reconstruction of fMRI data acquired at high field using* k-t* FOCUSS varies greatly with sampling scheme and thus the sampling scheme must be selected appropriately. Information in both *k*-space low and high frequency regions is important for better reconstruction performance and preservation of signal details, respectively, and thus sampling schemes that achieve a certain balance between the two must be selected for the application of CS to non-EPI fMRI data. The two* k-t* FOCUSS algorithms, temporal FT and KLT, showed good reconstruction results overall with effective suppression of downsampling artifacts and improved spatial resolution and thus are good candidates for CS in high field non-EPI fMRI studies. The application of CS to fMRI has great potential in practice for improvement of temporal resolution and/or spatial coverage.

## Figures and Tables

**Algorithm 1:**
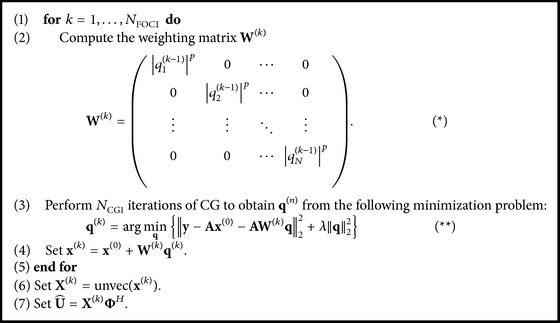
$\hat{U}$ = ktFOCUSS(*p*_1_, *λ*_1_, *N*_CG1_, *N*_FOC1_, Φ, **x** ^(0)^).

**Algorithm 2:**
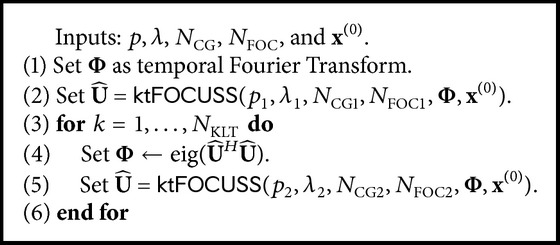
*k*-*t* FOCUSS with Karhunen-Loéve transform.

**Table 1:** ROC curve AUC values from fMRI maps of bSSFP time-series data with PC angle of 180° of a representative rat.

4x Down Center (control)		CS			
		Pattern_R_	Pattern_G_	Pattern_GR_	Pattern_GRC1_
0.9794	Algorithm 1	0.8943	0.9800	0.9795	0.9827
	Algorithm 2	0.8639	0.9726	0.9714	0.9825

**Table 2:** Reconstruction speed of *k*-*t* FOCUSS algorithms on downsampled time-series data using Pattern_GRC1_ of a representative rat.

	Algorithm 1		Algorithm 2		
	Single FT-CG iteration	Total time	Covariance calculation	Single KLT-CG iteration	Total time
bSSFP PC2	0.46 s	41.35 s	27.81 s	0.64 s	77.26 s
GRE	0.24 s	21.29 s	14.27 s	0.29 s	34.98 s

^*∗*^FT: Fourier transform, CG: conjugate gradient method, and KLT: Karhunen-Loéve transform.

^*∗∗*^Calculated on PC (Windows 7), CPU: 3.30 GHz, RAM: 4.00 GB.

**Table 3:** ROC curve AUC values from reconstructed fMRI maps using *k*-*t* FOCUSS algorithms and pairwise sampling of PE lines with Pattern_GRC1_ on bSSFP time-series data with PC angle of 180° of a representative rat.

	Algorithm 1	Algorithm 2
Pairwise PE Pattern_GRC1_	0.9810	0.9736

## Appendix

### Review of *k*-*t* FOCUSS

*k-t FOCUSS with Temporal Fourier Transform*.* k-t* FOCUSS is a recent CS algorithm developed for the reconstruction of dynamic image data [16, 18]. As the name indicates, it is based on FOCal Underdetermined System Solver (FOCUSS) algorithm and utilizes random sampling in the *k*-*t* domain [16, 43, 44]. Here, the basic structure of the algorithm will be speculated. For simplicity, only the case of Cartesian *k*-space trajectory will be discussed, with downsampling only in the phase-encoding direction and full sampling in the frequency-encoding direction.

In a discrete setup, the measurement-signal relationship at time *t* is

(A.1) $$\begin{array}{c} y_{t}=Fu_{t},\;t=1,\ldots ,T, \end{array}$$

where **F** ∈ *ℂ* ^*m*×*n*^ denotes a* downsampled* Fourier transform (FT) along the phase-encoding direction, **y**_*t*_ ∈ *ℂ* ^*m*^ denotes the *k* space measurement vectors, **u**_*t*_ ∈ *ℂ* ^*n*^ denotes the image vector at time *t*, and *T* denotes the number of time frames. If image content varies periodically over time, we can sparsify its temporal variation using *T* × *T* FT matrix Φ such that the corresponding coefficients {**x**_*t*_} become sparse:

(A.2) $$\begin{array}{c} U≜\left[u_{1},u_{2},\ldots ,u_{T}\right]=\left[x_{1},x_{2},\ldots ,x_{T}\right]\Phi^{H}=X\Phi^{H}, \end{array}$$

where **X**≜[**x**_1_, **x**_2_,…, **x**_*T*_].

Then, the measurement-signal relationship becomes

(A.3) $$\begin{array}{c} Y≜\left[y_{1},y_{2},\ldots ,y_{T}\right]=FX\Phi^{H}. \end{array}$$

Using the property vec(**A** **B** **C**) = (**A** ⊗ **C** ^*H*^)vec(**B**), where vec(·) denotes columnwise vectorization operation, we have

(A.4) $$\begin{array}{c} y=\left(F⊗\Phi \right)x=Ax, \end{array}$$

where **y** = vec(**Y**) ∈ *ℂ* ^*mT*^ and **x** = vec(**X**) ∈ *ℂ* ^*nT*^. In* k-t* FOCUSS, the unknown signal **x** is further decomposed as

(A.5) $$\begin{array}{c} x=x_{0}+\Delta x, \end{array}$$

where **x**_0_ denotes a predicted signal (such as temporal mean) and Δ**x** denotes residual signal that needs to be reconstructed using CS. Accordingly, the CS formulation is given by

(A.6) $$\begin{array}{c} min{\left\|\Delta x\right\|}_{1}\;s.t.\;\;{\left\|y-Ax_{0}-A\Delta x\right\|}_{2}\leq \epsilon . \end{array}$$

As an optimization method for (A.6),* k-t* FOCUSS employs weighted-*l*_2_ minimization or FOCUSS algorithm by converting Δ**x** = **W** **q**, which provides the following unconstrained form of cost function:

(A.7) $$\begin{array}{c} C\left(q\right)={\left\|y-Ax_{0}-AWq\right\|}_{2}^{2}+\lambda {\left\|q\right\|}_{2}^{2}, \end{array}$$

where

(A.8) $$\begin{array}{c} W=\left(\begin{array}{cccc} {\left|{\bar{q}}_{1}\right|}^{p} & 0 & \cdots & 0\\ 0 & {\left|{\bar{q}}_{2}\right|}^{p} & \cdots & 0\\ \vdots & \vdots & \ddots & \vdots \\ 0 & 0 & \cdots & {\left|{\bar{q}}_{N}\right|}^{p} \end{array}\right),\;\frac{1}{2}\leq p\leq 1.\; \end{array}$$

Here, $\bar{q}=[{\bar{q}}_{1},\ldots ,{\bar{q}}_{N}]$ denotes the estimated signal **x** from the previous iteration with *N* = *nT*. Then, the optimal solution of the problem is found by calculating the following:

(A.9) $$\begin{array}{c} x=x_{0}+\Theta A^{H}{\left(A\Theta A^{H}+\lambda I\right)}^{-1}\left(y-Ax_{0}\right), \end{array}$$

where Θ = **W** **W** ^*H*^. Since the direct calculation of (A.9) is computationally demanding due to the matrix inversion of a large size matrix, conjugate gradient (CG) method is used to minimize the cost function (A.7).

A generic form of the implementation of* k-t* FOCUSS that utilizes temporal FT Φ is summarized in Algorithm 1.

*k-t FOCUSS with KLT*. Even though* k-t* FOCUSS has been developed as above utilizing temporal FT as Φ in (A.3), other temporal transformations could also be used to efficiently sparsify the signal [16]. One example is the utilization of Karhunen-Loéve transform (KLT), which is also known as principal component analysis (PCA) [45].

KLT or PCA is a data-dependent mathematical procedure that uses an orthogonal transformation to convert possibly correlated elements of the data into a set of linearly uncorrelated components called “principal components.” The transformation leads to a result in which the first principal component accounts for the largest possible variance of the data, and each succeeding component has the next largest variance possible under the restriction that it is orthogonal (i.e., uncorrelated) to the preceding components. The transform is known to compact most of the energy into a small number of expansion coefficients [45] and thus is ideal in CS perspective [16].

The application of KLT within* k-t* FOCUSS requires calculation of a covariance matrix from a trained dataset. In this paper, the result from* k-t* FOCUSS that utilizes temporal FT using Algorithm 1 is used for the calculation of covariance matrix. Once the training dataset is defined, the covariance matrix is constructed as follows:

(A.10) $$\begin{array}{c} C={\hat{U}}^{H}\hat{U}. \end{array}$$

Then, the eigenvectors of **C** can be used for the KL transform; that is,

(A.11) $$\begin{array}{c} \Phi ⟵eig\left(C\right). \end{array}$$

After updating Φ, we can perform additional* k-t* FOCUSS iterations. The algorithm is summarized in Algorithm 2.

## References

[1] Ogawa S., Lee T. M., Kay A. R., Tank D. W. (1990). Brain magnetic resonance imaging with contrast dependent on blood oxygenation. *Proceedings of the National Academy of Sciences of the United States of America*.

[2] Bowen C., Menon R., Gati J. High field balanced-SSFP fMRI: a BOLD technique with excellent tissue sensitivity and superior large vessel suppression.

[3] Frahm J., Bruhn H., Merboldt K. D., Hänicke W. (1992). Dynamic MR imaging of human brain oxygenation during rest and photic stimulation. *Journal of Magnetic Resonance Imaging*.

[4] Henkin R. I., Levy L. M. (2001). Lateralization of brain activation to imagination and smell of odors using functional magnetic resonance imaging (fMRI): left hemispheric localization of pleasant and right hemispheric localization of unpleasant odors. *Journal of Computer Assisted Tomography*.

[5] Jin H. L., Dumoulin S. O., Saritas E. U. (2008). Full-brain coverage and high-resolution imaging capabilities of passband b-SSFP fMRI at 3T. *Magnetic Resonance in Medicine*.

[6] Miller K. L., Smith S. M., Jezzard P., Wiggins G. C., Wiggins C. J. (2007). Signal and noise characteristics of SSFP FMRI: a comparison with GRE at multiple field strengths. *NeuroImage*.

[7] Park S.-H., Kim T., Wang P., Kim S.-G. (2011). Sensitivity and specificity of high-resolution balanced steady-state free precession fMRI at high field of 9.4T. *NeuroImage*.

[8] Wowk B., McIntyre M. C., Saunders J. K. (1997). k-Space detection and correction of physiological artifacts in fMRI. *Magnetic Resonance in Medicine*.

[9] Zhong K., Leupold J., Hennig J., Speck O. (2007). Systematic investigation of balanced steady-state free precession for functional MRI in the human visual cortex at 3 Tesla. *Magnetic Resonance in Medicine*.

[10] Griswold M. A., Jakob P. M., Heidemann R. M. (2002). Generalized autocalibrating partially parallel acquisitions (GRAPPA). *Magnetic Resonance in Medicine*.

[11] Pruessmann K., Weiger M., Scheidegger M., Boesiger P. (1999). SENSE: sensitivity encoding for fast MRI. *Magnetic Resonance in Medicine*.

[12] Sodickson D. K., Manning W. J. (1997). Simultaneous acquisition of spatial harmonics (SMASH): fast imaging with radiofrequency coil arrays. *Magnetic Resonance in Medicine*.

[13] Lustig M., Donoho D., Pauly J. M. (2007). Sparse MRI: the application of compressed sensing for rapid MR imaging. *Magnetic Resonance in Medicine*.

[14] Candés E. J., Romberg J., Tao T. (2006). Robust uncertainty principles: exact signal reconstruction from highly incomplete frequency information. *IEEE Transactions on Information Theory*.

[15] Donoho D. L. (2006). Compressed sensing. *IEEE Transactions on Information Theory*.

[16] Jung H., Ye J. C., Kim E. Y. (2007). Improved *k*−*t* BLAST and *k*−*t* SENSE using FOCUSS. *Physics in Medicine and Biology*.

[17] Gamper U., Boesiger P., Kozerke S. (2008). Compressed sensing in dynamic MRI. *Magnetic Resonance in Medicine*.

[18] Jung H., Sung K., Nayak K. S., Kim E. Y., Ye J. C. (2009). K-t FOCUSS: a general compressed sensing framework for high resolution dynamic MRI. *Magnetic Resonance in Medicine*.

[19] Jeromin O., Pattichis M. S., Calhoun V. D. (2012). Optimal compressed sensing reconstructions of fMRI using 2D deterministic and stochastic sampling geometries. *BioMedical Engineering Online*.

[20] Jung H., Ye J. Performance evaluation of accelerated functional MRI acquisition using compressed sensing.

[21] Holland D. J., Liu C., Song X. (2013). Compressed sensing reconstruction improves sensitivity of variable density spiral fMRI. *Magnetic Resonance in Medicine*.

[22] Zong X., Lee J., John Poplawsky A., Kim S. G., Ye J. C. (2014). Compressed sensing fMRI using gradient-recalled echo and EPI sequences. *NeuroImage*.

[23] Silva A. C., Lee S.-P., Yang G., Ladecola C., Kim S.-G. (1999). Simultaneous blood oxygenation level-dependent and cerebral blood flow functional magnetic resonance imaging during forepaw stimulation in the rat. *Journal of Cerebral Blood Flow and Metabolism*.

[24] Bottomley P. A. (1987). Spatial localization in NMR spectroscopy *in vivo*. *Annals of the New York Academy of Sciences*.

[25] Chen L., Samsonov A., Dibella E. V. R. (2011). A Framework for generalized reference image reconstruction methods including HYPR-LR, PR-FOCUSS, and k-t FOCUSS. *Journal of Magnetic Resonance Imaging*.

[26] Sorenson J. A., Wang X. (1996). ROC methods for evaluation of fMRI techniques. *Magnetic Resonance in Medicine*.

[27] Du Q., Fowler J. E. (2007). Hyperspectral image compression using JPEG2000 and principal component analysis. *IEEE Geoscience and Remote Sensing Letters*.

[28] Penna B., Tillo T., Magli E., Olmo G. (2007). Transform coding techniques for lossy hyperspectral data compression. *IEEE Transactions on Geoscience and Remote Sensing*.

[29] Scheffler K., Lehnhardt S. (2003). Principles and applications of balanced SSFP techniques. *European Radiology*.

[30] Miller K. L., Tijssen R. H. N., Stikov N., Okell T. W. (2011). Steady-state MRI: methods for neuroimaging. *Imaging in Medicine*.

[31] Chen S. S., Donoho D. L., Saunders M. A. (1998). Atomic decomposition by basis pursuit. *SIAM Journal on Scientific Computing*.

[32] Boufounos P., Duarte M. F., Baraniuk R. G. Sparse signal reconstruction from noisy compressive measurements using cross validation.

[33] Ward R. (2009). Compressed sensing with cross validation. *IEEE Transactions on Information Theory*.

[34] Angelosante D., Giannakis G. B., Grossi E. Compressed sensing of time-varying signals.

[35] Bieri O., Markl M., Scheffler K. (2005). Analysis and compensation of eddy currents in balanced SSFP. *Magnetic Resonance in Medicine*.

[36] Nguyen H. M., Glover G. H. (2013). A modified generalized series approach: application to sparsely sampled FMRI. *IEEE Transactions on Biomedical Engineering*.

[37] Friston K. J., Jezzard P., Turner R. (1993). Analysis of functional MRI time-series. *Human Brain Mapping*.

[38] Boynton G. M., Engel S. A., Glover G. H., Heeger D. J. (1996). Linear systems analysis of functional magnetic resonance imaging in human V1. *The Journal of Neuroscience*.

[39] Woolrich M. W., Ripley B. D., Brady M., Smith S. M. (2001). Temporal autocorrelation in univariate linear modeling of FMRI data. *NeuroImage*.

[40] Lingala S. G., Hu Y., Dibella E., Jacob M. (2011). Accelerated dynamic MRI exploiting sparsity and low-rank structure: k-t SLR. *IEEE Transactions on Medical Imaging*.

[41] Asif M. S., Hamilton L., Brummer M., Romberg J. (2013). Motion-adaptive spatio-temporal regularization for accelerated dynamic MRI. *Magnetic Resonance in Medicine*.

[42] Yoon H., Kim K., Kim D., Bresler Y., Ye J. (2014). Motion adaptive patch-based low-rank approach for compressed sensing cardiac cine MRI. *IEEE Transactions on Medical Imaging*.

[43] Gorodnitsky I. F., George J. S., Rao B. D. (1995). Neuromagnetic source imaging with FOCUSS: a recursive weighted minimum norm algorithm. *Electroencephalography and Clinical Neurophysiology*.

[44] Gorodnitsky I., Rao B. (1997). Sparse signal reconstruction from limited data using FOCUSS: a re-weighted minimum norm algorithm. *IEEE Transactions on Signal Processing*.

[45] Poor H. V. (1988). *An Introduction to Signal Detection and Estimation*.

